# Novel and recurrent *BRCA1/BRCA2* germline mutations in patients with breast/ovarian cancer: a series from the south of Tunisia

**DOI:** 10.1186/s12967-021-02772-y

**Published:** 2021-03-16

**Authors:** Dorra Ben Ayed-Guerfali, Wala Ben Kridis-Rejab, Nihel Ammous-Boukhris, Wajdi Ayadi, Slim Charfi, Afef Khanfir, Tahia Sellami-Boudawara, Mounir Frikha, Jamel Daoud, Raja Mokdad-Gargouri

**Affiliations:** 1 Center of Biotechnology of Sfax, University of Sfax, Sidi Mansour Street Km 6, BP 1177, 3038 Sfax, Tunisia; 2 Department of Oncology, Habib Bourguiba Hospital, Sfax, Tunisia; 3 Department of Anatomo-pathology, Habib Bourguiba Hospital, Sfax, Tunisia; 4 Department of Radiotherapy, Habib Bourguiba Hospital, Sfax, Tunisia

**Keywords:** Breast cancer, Ovarian cancer, BRCA1, BRCA2, Germline mutation, Genetic testing

## Abstract

**Background:**

The incidence of breast cancer (BC) and/or ovarian cancer (OC) is increasing in Tunisia especially in young women and mostly those with family history. However, the spectrum of *BRCA* mutations remains little explored in Tunisian patients in particular in the southern region.

**Methods:**

We sequenced the entire coding regions of *BRCA1*and *BRCA2* genes using next generation sequencing (NGS) in 134 selected patients with BC and/or OC.

**Results:**

Among the 134 patients, 19 (14.17%) carried pathogenic mutations (10 are *BRCA1* mutation carriers and 9 are *BRCA2* mutation carriers) that are mainly frameshift index (76.9%). Interestingly, 5 out of the 13 variants (38.46%) were found at least twice in unrelated patients, as the c.1310-1313 delAAGA in *BRCA2* and the c.5030_5033 delCTAA that has been identified in 4/98 BC patients and in 3/15 OC patients from unrelated families with strong history of cancer. Besides recurrent mutations, 6 variant (4 in *BRCA1* and 2 in *BRCA2*) were not reported previously. Furthermore, 3 unrelated patients carried the VUS c.9976A > T, (K3326*) in *BRCA2* exon 27. *BRCA* carriers correlated significantly with tumor site (p = 0.029) and TNBC cases (p = 0.008). In the groups of patients aged between 31 and 40, and 41–50 years, *BRCA1* mutations occurred more frequently in patients with OC than those with BC, and conversely *BRCA2* carriers are mostly affected with BC (p = 0.001, and p = 0.044 respectively).

**Conclusions:**

The overall frequency of the BRCA germline mutations was 14.17% in patients with high risk of breast/ovarian cancer. We identified recurrent mutations as the c.1310_1313 delAAGA in *BRCA2* gene and the c.5030_5033 delCTAA in *BRCA1* gene that were found in 4% and 20% of familial BC and OC respectively. Our data will contribute in the implementation of genetic counseling and testing for families with high-risk of BC and/or OC.

## Background

Breast Cancer (BC) is the most prevalent cancer worldwide and the second leading cause of death by cancer in women [1, 2]. In Tunisia, the incidence of BC is 27.1/100.000 per year affecting more often young women (< 35 years), and with more aggressive clinical behavior [3, 4]. Approximately 5 to 10% of BC patients harbor germline mutations that predispose to this malignancy at earlier onset compared to the general population [5, 6].

Evidently, the BC susceptibility genes *BRCA1* (MIM# 113705) and *BRCA2* (MIM# 600185) are tumor suppressor genes that play a key role in DNA repair through the homologous recombination pathway [7, 8]. Therefore, loss of the *BRCA* function results to an inefficient DNA repair process that increases the mutation rates and thus contributes to tumor development [9, 10]. Indeed, women carrying pathogenic germline mutations in *BRCA1*/*BRCA2* genes have an increased risk of BOC compared to those with wild type *BRCA* genes [11–13]. Screening of *BRCA1*/*BRCA2* genes for the identification of pathogenic mutations is essential to provide genetic counseling to the members of affected families, for medical follow-up, and for the targeted therapy selection which is based on the inhibitor of poly-ADP-ribose polymerase inhibitor namely the Olaparib [14]. Despite the importance of the mutational spectrum of *BRCA1/BRCA2* genes, only few studies using Sanger DNA sequencing, investigated Tunisian patients with HBOC. In a recent study, Laitamn et al., reported all the pathogenic variants in both *BRCA1* and *BRCA2* genes in the Middle East, North Africa, and Southern Europe [15]. The authors identified 232 and 239 pathogenic sequence variants in *BRCA1* and *BRCA2*, respectively that include only few variants that were found in Tunisian patients [15].

Besides, Riahi et al., reported twelve pathogenic mutations (25%); among them, three were found in *BRCA*1 (c.211dupA, c.5266dupC and c.1504_1508delTTAAA) and two were novel mutations detected in *BRCA*2 (c.1313dupT and c.7654dupT). The study was carried out on 92 families and was performed by direct sequencing [16]. Furthermore, two other studies reported deleterious mutations of the *BRCA1/BRCA2* genes in 19% and 18% of familial patients from the north region of Tunisia respectively [17, 18]. Moreover, Fourati et al. by targeting the exons 5, 11, and 20 of the *BRCA1* gene and exons 10 and 11 of the *BRCA2* gene, showed that among 66 patients, only 12 patients (18%) had deleterious mutations in the *BRCA1* or *BRCA2* genes [19].

The study of Mahfoudh et al., included 16 Tunisian high-risk BC families and they were screened for only *BRCA1* gene. The authors showed that the prevalence of *BRCA1* carriers was 37.5% and identified 3 truncating mutations (c.916 delTT, c.3450 delCAAG, c.5382 insC) and one splice site mutation c.212 + 2insG [20].

Technological advances in DNA sequencing have improved and facilitated the screening procedure of *BRCA1/BRCA2* genes since the large size of these genes and the absence of hotspots regions make this procedure expensive and time-consuming. In fact, NGS has resolved these problems and allows, henceforth the detection of mutations with higher sensibility [21–23]. Our study is the first using NGS to screen the whole exons of *BRCA1/BRCA2* genes in 134 selected patients with high risk of BOC in the south region of Tunisia.

## Methods

### Patients

A total of 134 patients were selected (between 2016 and 2019) among them 113 patients with HBOC (98 cases) and/or OC (15 cases) that meet one of the following criteria: (1) Presence of at least three related first or second-degree BC cases; (2) BC in young patients aged less than 35 years, (3) Presence of male BC among the first or second-degree relatives, (4) Presence of at least two cases of BC or OC, regardless of age, and at least one case of prostate cancer in a related first or second degree patient.

In addition, 21 cases without evidence of family history were included in this study, 12 were diagnosed with BC at the age ≤ 40, and 9 patients diagnosed with OC at the age ≤ 60 years. All patients were recruited from the department of Medical Oncology of the CHU Habib Bourguiba of Sfax (Tunisia), and had provided written informed consent for participation in the research study and for the genetic testing.

### Genetic testing

Genomic DNA was isolated from 0.2 mL of peripheral blood of the proband from each selected family using the “QIAamp DNA Blood Mini kit” (Qiagen), following the manufacturer’s instructions. Isolated DNA was quantified by Qubit 3.0 Fluorometric quantitation (Thermo Fisher Scientific). The *BRCA/BRCA2* germline mutations were detected using the next-generation sequencing approach (NGS). Briefly, 50 ng of genomic DNA from each sample was used to prepare library using the “AmpliSeq™BRCA Panel”, “AmpliSeq™Library Plus” and “AmpliSeq™CD Indexes” according to the protocol provided by Illumina. The adaptors and sample unique DNA barcodes were incorporated into the amplified amplicons with a second PCR. Libraries were quantified with the Qubit^®^ dsDNA HS Assay Kit (Life Technologies).The DNA library was pooled and prepared for sequencing using the Illumina MiSeq sequencer with Miseq Reagent Kit v2 (300-cycles) (Illumina, San Diego, CA) according to the manufacturer’s instructions to generate paired-end reads with a 151-bp read length. Reads were trimmed to remove low-quality sequences and then aligned to the human reference genome (GRCh37/hg19) using the Burrows-Wheeler alignment (BWA) package. The total PF Reads is 16,755,084 and the Q30 is 93.56%.

The *BRCA1* (NM_007300.3) and *BRCA2* (NM_000059.3) sequences from the National Center for Biotechnology Information (NCBI) database (http://www.ncbi.nlm.nih.gov) were used as reference. The NGS data was analyzed using the BaseSpace Variant Interpreter (https://basespace.illumina.com).

Sanger sequencing was performed to confirm the *BRCA1/BRCA2* pathogenic mutation identified by NGS (see Additional file 1: Fig.1 in Supplementary data). Sequencing primers were designed using Primer 5.0 software and provided on request. PCR products were purified using the QIAquick PCR Purification Kit (Qiagen) and labeled using the BigDye Terminator v3.1 Cycle Sequencing Kit (Applied Biosystems), and the sequence analysis was performed by BioEdit.

### Variant annotation and classification

We annotated the mutations using in silico prediction tools to evaluate the functional effects of the candidate variants: PolyPhen-2 (http://genetics.bwh.harvard.edu/pph2/), which predicts the effects of an amino acid substitution on the structure and function of a protein. The PolyPhen score represents the probability that a substitution is damaging, so values between 0.9 to 1 are more confidently predicted to be deleterious. The program sorting intolerant from tolerant (SIFT) predicts whether an amino acid substitution is likely to affect protein function based on sequence homology and the physicochemical similarity between the alternate amino acid. The score is the normalized probability that the amino acid change is deleterious if the score is < 0.05 and tolerated if the score is > 0.05. Mutation Taster (http://www.Mutationtaster.org/) was used to assess the impact of mutations on protein function.

### Statistical analysis

Associations between patient characteristics and presence of pathogenic *BRCA1/BRCA2* mutations were tested by Chi-square test. A significance level of p ≤ 0.05 was used in the analysis. All analyses were performed using SPSS (version 20.0).

## Results

### Patients characteristics

A total of 134 breast and/or ovarian cancer subjects were included in this study. The characteristics of the study group are presented in Table 1. Unilateral BC was diagnosed in 104 (77.6%) patients, 6 (4.5%) women had bilateral BC, 22 (16.4%) had OC, and 2 (1.5%) had both BC and OC. Age at diagnosis of BC ranged from 27 to 65 years (median: 42.78 years), and of OC ranged from 38 to 64 years (median: 50.37 years). For patients with BC only 9 (6.7%) were diagnosed before the age of 30 years and 55/134 (41%) before 40 years of age. In the group of patients with OC only 4 patients were diagnosed at an age ≤ 40 years. Family history of BC and/or OC was found in 113 (84.3%) patients. Among the 21 patients without family history of BC/OC, 12 were diagnosed with TNBC and 9 with OC (Table 1).

**Table 1:** Clinicopathological parameters and association with *BRCA1/BRCA2* carriers

	N (%)	Non carrier (%)	*BRCA1* carrier (%)	*BRCA2* carrier (%)	p
Family history					
Yes	113 (84.3)	95 (84)	9 (8)	9(8)	0.33
No	21 (15.7)	20 (95.2)	1 (4.8)	0 (0.00)	
Age					
≤ 30	9 (6.7)	7 (77.8)	1 (11.1)	1(11.1)	0.433
31–40	55 (41)	45 (81.8)	7 (12.7)	3 (5.5)	
41–50	33 (24.6)	29 (87.9)	2 (6.1)	2 (6.1)	
≥ 51	37 (27.6)	34 (91.9)	0 (0.00)	3 (8.1)	
Tumor site					
Unilateral BC	104 (77.6)	92 (88.5)	4 (3.8)	8 (7.7)	***0.029***
Bilateral BC	6 (4.5)	4 (66.7)	1 (16.7)	1 (16.7)	
OC	22 (16.4)	18 (81.8)	4 (18.2)	0 (0.00)	
OC/BC	2 (1.5)	1(50)	1 (50)	0 (0.00)	
Tumor grade					
SBR II	82 (73.2)	73 (89)	4 (4.9)	5 (6.1)	0.41
SBR III	30 (26.8)	24 (80)	2 (6.7)	4 (13.3)	
T-stage					
T0–T1	11 (8.2)	9 (81.8)	2 (18.2)	0 (0.00)	0.16
T2	63 (47)	56 (88.9)	2 (3.2)	5(7.9)	
T3	49 (36.6)	41 (83.7)	6 (12.2)	2 (4.1)	
T4	11 (8.2)	9 (81.8)	0 (0.00)	2 (18.2)	
N-stage					
No	54 (40.3)	48 (88.9)	3 (5.6)	3 (5.6)	0.69
N1–N2	80 (59.7)	67 (83.8)	7 (8.8)	6 (7.5)	
M					
M0	120 (89.6)	105 (87.5)	7 (5.8)	8 (6.7)	0.107
M1	14 (10.4)	10 (71.4)	3 (21.4)	1 (7.1)	
Triple negative					
No	84 (76.4)	75 (89.3)	1 (1.2)	8 (9.5)	***0.008***
Yes	26 (23.6)	21 (80.8)	4 (15.4)	1 (3.8)	

### *BRCA1/BRCA2* germline pathogenic mutations

**Fig. 1:**
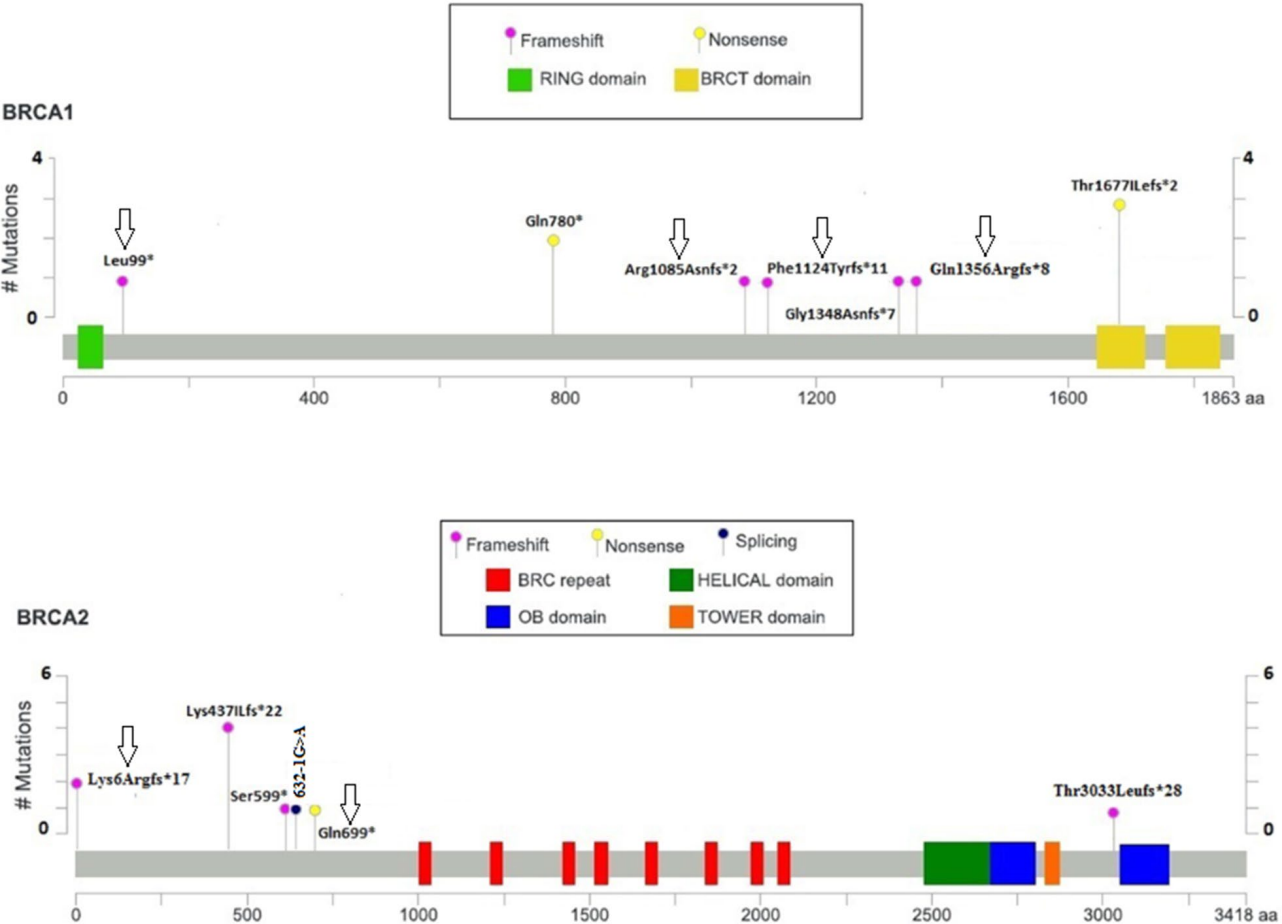
Schematic diagram of *BRCA1* and *BRCA2* proteins with the position of all identified pathogenic variants. Novel variants are marked with arrow

**Fig. 2:**
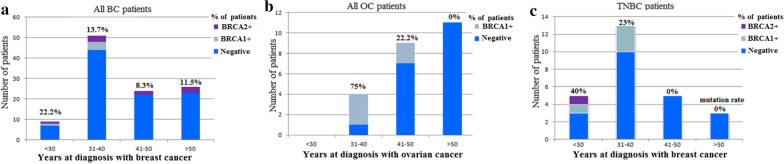
*BRCA* mutation rate (%) in all BC (**a**), in all OC (**b**), and in TNBC (**c**) patients divided according to age at diagnosis (≤ 30, 31–40, 41–50, and ≥ 51 years old)

Pathogenic *BRCA1/BRCA2* germline mutations were identified in 14.17% (19/134) of the patients (Fig. 1, Table 2). Among 113 patients with strong family history for breast/ovarian cancer (HBOC), 18 (15.9%) were positive for *heterozygous BRCA* mutations (9 in *BRCA1* and 9 in *BRCA2*). In the group of patients without evidence of HBOC, including 12 patients with TNBC and 9 patients with OC, only one patient 4.8% (1/21) with OC carried the c.2338C > T (Gln780X) pathogenic *BRCA1* mutation and 2 cases (1 TNBC and 1 OC) carried the VUS c.9976A > T (Lys3326X) in *BRCA2* exon 27 (Fig. 1, Tables 2,4). Most of the *BRCA* mutations were pathogenic (class 2 to 6) and 76.9% of the mutation (10/13) were frameshift deletion, only 1 was frameshift duplication, 2 nonsens mutations and one splice site mutation (Table 2, Fig. 1). Among the 13 mutations detected, 5 were identified at least twice in unrelated patients. Notably, the frameshift mutation c.1310_1313delAAGA of the *BRCA2* gene was detected in 4 unrelated BC patients, and the c.17-20 delAAGA in 2 unrelated young patients with strong family history of BC. In *BRCA1*, the c.5030_5033delCTAA was found in 3 unrelated young patients (≤ 40 years) diagnosed with OC (2 without and 1 with BC), and the c.2338C > T mutation was detected in 2 unrelated patients with OC only. Interestingly, our data indicated that the c.1310_1313delAAGA of the *BRCA2* gene, and the c.5030_5033delCTAA of the *BRCA1* gene were found in 4% (4/98) and in 20% (3/15) of familial BC and of early onset familial OC respectively. We also detected the c.632-1G > A mutation in the splice acceptor site in 2 patients with BC (Table 2, Fig. 2). It is interesting to note that the 2 unrelated patients with the c.632-1G > A variant, carried also the pathogenic mutation c.1310_1313delAAGA in *BRCA2*. Our study outlined 6 out of 13 (46%) novel pathogenic *BRCA* mutations, according to the BIC and ClinVar databases.

**Table 2:** Pathogenic *BRCA1/BRCA2* mutations identified in breast/ovarian cancer patients

Gene	DNA change	Protein change	Total read depth	Variant read frequency	BIC, ClinVar	Class	Tumor site	Age	Fq
*BRCA1*	c.4067-4071 delAAGAA	GLn1356Argfs*8	798	0.5293	NR	5	Bil BC	30	1
	c.5030_5033delCTAA	Thr1677Ilefs*2	1609	0.5320	R	6	BC, OC, BC/OC	38,39,40	3
	c.296_297delTG	Leu99*	429	0.4779	NR	–	BC	43	1
	c.2338C > T	Gln780*	626	0.5064	R	5	OC, OC	46,48	2
	c.3254delG	Arg1085Asnfs*2	796	0.4950	NR	5	BC	33	1
	c.3364_3370 dupACAGATT	Phe1124Tyrfs*11	421	0.4676	NR	–	BC	40	1
	c.4041_4042delAG	Gly1348Asnfs*7	925	0.4941	R	4	BC	40	1
*BRCA2*	c.17-20 delAAGA	Lys6Argfs*17	1238	0.4651	NR	5	BC	39	2
	c.1310-1313 delAAGA	Lys437Ilefs*22	2206	0.4955	R	5	BC	57,42, 27,58	4
	c.1976_1800 delCTTAT	Ser599*	2476	0.5202	R	4	BC	37	1
	c.2095C > T	Gln699*	709	0.5162	NR	5	BC	43	1
	c.9097delA	Thr3033Leufs*28	1614	0.5165	R	5	BC	63	1
	c.632-1G > A	–	826	0.5133	R	2	BC	27,57	2

BC: Breast cancer; OC: ovarian cancer; Bil BC: bilateral breast vancer; Fq: frequency of the identified mutation in BRCA+ patients; R: reported; NR: not Reported

### Association of BRCA carriers and clinico-pathological parameters

In BC, there is a significant association between Triple Negative status and *BRCA* mutations. Among the 26 TNBC cases, 5 (19.23%) carried *BRCA* mutations and 4 (80%) were *BRCA1* mutation carriers (p = 0.008, Table 1). Additionally, patients with OC (with or without BC) carried only mutations in *BRCA1* gene as opposed to *BRCA2* gene (5/24 vs 0/24), whereas *BRCA2* mutations were more frequently detected in patients with only BC (9/110 vs 5/110, p = 0.029, Table 1). No other associations were observed with clinico-pathological parameters (Table 1).

On the other hand, for patients diagnosed with BC at an age ≤ 30 years, the *BRCA* mutation rate was 22.2% (2/9), and none of the patients was diagnosed with OC at an age ≤ 30 years in our series. In the group of patients between 31 and 40 years, *BRCA* mutation was detected in 13.72% (7/51) and in 75% (3/4) of women diagnosed with BC and OC respectively (p = 0.001, Fig. 2a, b; Table 3). Concerning women aged from 41 to ≤ 50 years old diagnosed with BC or OC, 8.33% (2/24) and 22.2% (2/9) carried a *BRCA* mutation respectively (p = 0.044, Fig. 2a, b; Table 3). None of OC patients over 51 years carried *BRCA* mutation while 11.5% (3/26) of BC patients were *BRCA2* carriers (Fig. 2a, b; Table 3). The mutation rate for the *BRCA* gene in patients with only OC was 18.2% (4/22), all were *BRCA1* carriers and only one OC patient carried the variant K3326X in the *BRCA2* gene (Tables 1, 3). Furthermore, in young patients (≤ 30 years), 40% (2/5) of TNBC carried *BRCA* mutation while in the group of TNBC patients aged from 31 to 40 years old, *BRCA* mutations occurred more frequently in *BRCA1* than in *BRCA2* gene (3/13, (23%) vs 0/13 (0%), (p = 0.041, Fig. 2c, Table 3).

**Table 3:** Association of *BRCA1/BRCA2* carriers with tumor site and triple negative status in patients divided according to the age at diagnosis

Age	Tumor Site	Non carriers (%)	*BRCA1* carriers (%)	*BRCA2* carriers (%)	p
≤ 30	BC	7 (77.8)	1 (11.1)	1 (11.1)	**–**
	OC	0 (0)	0 (0)	0 (0)	
31–40	BC	44 (86.3)	4(7.8)	3 (5.9)	***0.001***
	OC	1 (25)	3 (75)	0 (0)	
41–50	BC	22 (91.7)	0 (0)	2 (8.3)	***0.044***
	OC	7 (77.8)	2 (22.2)	0 (0)	
≥ 51	BC	23 (88.5)	0 (0)	3 (11.5)	0.24
	OC	11 (100)	0 (0)	0 (0)	

Age	TN	Non carriers (%)	*BRCA1* carriers (%)	*BRCA2* carriers (%)	p
≤ 30	No	4 (100)	0 (0)	0 (0)	0.358
	Yes	3 (60)	1 (20)	1 (20)	
31–40	No	34 (89.5)	1 (2.6)	3 (7.9)	***0.041***
	Yes	10 (76.9)	3 (23.1)	0 (0)	
41–50	No	17 (89.5)	0 (0)	2 (10.5)	0.449
	Yes	5 (100)	0(0)	0 (0)	
≥ 51	No	20(87)	0 (0)	3 (13)	0.506
	Yes	3 (100)	0 (0)	0 (0)	

BC: Breast cancer; OC: ovarian cancer (with or without breast cancer); Yes: TN (ER−/PR−/Her2−); No: others (ER−/PR−/Her2+, ER+/PR+/Her2+, ER+/PR+/Her2−)

### Variants of uncertain significance in BRCA1/BRCA2 genes

In addition to deleterious *BRCA1/BRCA2* mutations, 10 VUS (6 in *BRCA1* and 4 in *BRCA2*) were predicted to be pathogenic by PolyPhen, SIFT or Mutation Taster (Table 4). Each VUS was identified in more than one patient and the c.9976A > T in exon 27 of the *BRCA2* gene was detected in 3 unrelated patients, 2 with BC diagnosed at an age ≤ 40 years and 1 patient with OC (Table 4). All VUS have been reported in the BIC database except the variant N550H in *BRCA1* exon 11.

**Table 4:** List of the uncertain significant variants (VUS)

Variants	Gene	Exon	AA change	Clinical importance (BIC)	Functional prediction		
					PolyPhen	SIFT	Mutation taster
c.293G > C	*BRCA1*	5	G98A	Unknown	PD	Del	DC
c.397 C > T	*//*	6	R 133C	//	PD	Del	DC
c.536A > G	*//*	7	Y179C	//	PD	Del	DC
c.1067A > G	*//*	11	Q356R	//	PD	D	Pol
c.1648A > C	*//*	11	N550H	NA	PD	D	Pol
c.5117G > C	*//*	17	G1706A	//	B	Del	DC
c.6100 C > T	*BRCA2*	11	R2034C	//	PD	D	Pol
c.7397T > C	*//*	14	V2466A	No	PD	D	Pol
c.7712A > G	*//*	16	E2571G	//	PD	D	DC
c.9976A > T	*//*	27	K3326*	LB	NA	NA	DC

PD: Probably deleterious; D: deleterious; DC: disease causing; Pol: polymorphism; LB: Likely Benign; NA: not available

## Discussion

*BRCA1* and *BRCA2* have key roles in the development of breast/ovarian cancer [9, 10]. The prevalence of *BRCA1/BRCA2* mutations varies in different populations due to founder mutation effect [15, 24–28]. Genetic testing of patients with family history for breast/ovarian cancer have become standard clinical management in Western countries, however, in Tunisia studies of *BRCA*-associated breast/ovarian cancer remain less investigated. Clearly, the majority of studies that reported the spectrum of *BRCA* mutations in Tunisia included a small number of patients and used Sanger method for DNA sequencing [16–20]. Riahi et al. reported that the rate of *BRCA* mutations in patients from the north of Tunisia was 25% (12/48) [16]. Furthermore, in another study, it was showed that deleterious *BRCA1* mutations were detected in 37% (6/16) of patients selected from high-risk breast cancer families [20]. In this study, we used NGS to determine the *BRCA* mutation rates in high risk breast and/or ovarian cancer in patients from the south of Tunisia. Thirteen truncating mutations and one splice acceptor mutation were identified in 19 among 134 patients (14.17%). The rate of *BRCA1* mutations found in this study (7.46%, 10/134) was lower than those reported in previous studies. In fact, in a review of the literature, Cherbal et al., reported that in breast/ovarian cancer families from Algeria, Morocco and Tunisia, *BRCA1* was mutated in 17.43% of cases (34/195) and that the c.798_799delTT was a recurrent mutation [29]. In our study none of the previously reported mutations in North African patients was identified except for the c.1310_1313delAAGA in *BRCA2*. Indeed, this mutation was detected in 4% (4/98) of patients from unrelated families with high risk of BC and among 9 *BRCA2* carriers, 4 (44.4%) had the c.1310_1313delAAGA mutation suggesting that it might be recurrent in our population. Our finding is in line with a previous study showing that the c.1310_1313delAAGA mutation was detected in 6% (4/66) of patients from the north of Tunisia [19]. Furthermore, it was reported that the c.1310_1313delAAGA mutation was detected in 11.4% (14/122) of patients from the North-East region of Morocco, and in one Algerian family among 10 carrying *BRCA* mutation [30, 31]. According to the Breast Cancer Information Core database (BIC; http://research.nhgri.nih.gov/bic/), this mutation was found in different European patients and was recorded several times in the French UMD-BRCA2 database and classified as founder mutation [32]. Furthermore, we found that 2 BC patients are double heterozygous carrying both the c.1310_1313delAAGA frameshift mutation and the splice site acceptor variant c.-632G < A. This splicing mutation has been reported two times in the ClinVar database therefore, and in the best of our knowledge this is the first report describing the association of the splice site and a frameshift mutation in the *BRCA2* gene in 2 unrelated patients with strong family history of BC.

The pathogenic mutation c.17-20 delAAGA (Lys6Xfs) in *BRCA2* exon 2 was detected in 2 unrelated young patients (39 year-old) with strong family history of BC. This mutation has not been reported previously in the ClinVar and BIC databases.

We also detected in the *BRCA2 *exon 27, the c.9976A > T (K3326X) in 3 unrelated patients, 2 were diagnosed with family BC at 28 and 36 years old and one patient with OC at 56 years old and without family history. The *BRCA2* variant (K3326X), was firstly interpreted as pathogenic, but its identification in control populations led to its classification as a benign polymorphism [33]. However, recent studies showed the association of K3326X variant with the risk of developing melanoma, pancreatic, breast and ovarian cancers [34–36]. Moreover, the large study of Meeks et al., provided evidence that the K3326X variant is associated with the risk of developing BC and OC independently of other pathogenic variants in *BRCA2* [37]. Altogether, it was suggested that the K3326X variant is not neutral and that it may be included in SNP panels for evaluating BC risk.

In *BRCA1*gene, we identified the c.5030_5033delCTAA mutation in 3 out of 15 (20%) unrelated patients with early onset  HBOC. This mutation has not been reported in North Africa but was found in patients from Greece, Italy, Jordan, Lebanon, Kuwait, and Saudia Arabia [15]. In addition, the nonsense mutation c.2338C > T was shared by 2 young patients (< 50 years-old) diagnosed with OC. The c. 2338C > T has been reported in Caucasian patients according to the BIC and ClinVar databases. Altogether, these findings may suggest ethnic and genetic associations between unrelated populations, or that these mutations occur in mutational hotspots region.

On the other hand, it is interesting to note that the *BRCA* mutation rate in young patients diagnosed with BC (age ≤ 30 years) was higher (22.2%) compared to older patients (13.7% (31–40), 8.3% (41–50), and 11.5% (≥ 51 years). The recent study of Bakkach et al.,  reported that the frequency of mutations among young patients (≤ 40 years) with family history of BC was 16.7% (5 out of 33 patients) suggesting that the young age for BC diagnosis seems to be strongly predictive of *BRCA* mutation status in Moroccan patients [38].

Of patients with OC (with and without BC), 20.83% (5/24) carried pathogenic variants. Mutation detection rates were 75% (3/4) for patients diagnosed between 31 and 40 years compared to only 22.2% (2/9) for women between 41 and 50 years. All patients over 51 years, carried no mutations in *BRCA1/BRCA2* genes.

It is well established that TNBC exhibit an aggressive behavior and has a worse prognosis [39]. In our cohort, among 110 patients with only BC, 26 (23.6%) were TN including 14 patients with family history of BC and 12 without evidence of family history of BOC. Five patients (5/26, 19.23%) carried *BRCA* mutations and 4 among them (80%) were *BRCA1* carriers. Consistent with our findings, in a previous study, the *BRCA1* mutation carriers had a higher incidence in TNBC than *BRCA2* mutation carriers [40–43]. Moreover, in a recent work, Toss et al., showed that in TNBC Italian patients, *BRCA* mutation prevalence was 22.6% (21.4% *BRCA1*) and that 64.2% are ≤ 30 years old, which is in line with our findings [44]. Furthermore, Mahfoudh et al., reported that the 5382insC mutation in *BRCA1* was detected in 25% (2/8) of Tunisian patients with TNBC [45]. In our study, none of the patients carried the *BRCA1* 5382insC mutation, however, in TNBC we detected 3 mutations in *BRCA1* (c.3254delG, c.3364_3370dupACAGATT, c.4067_4071delAAGAA), and no *BRCA2* pathogenic mutations were detected in TNBC cases except the VUS c.9976A > T identified in 2 unrelated patients (Additional file 2: Table 1 in Supplementary data).

It is important to note that previous studies used Sanger sequencing to identify BRCA mutations in Tunisian families with a history of BOC unlike the present study where the whole BRCA genes were screened by the NSG which allowed us to identify new mutations in Tunisian patients. However, some recent studies have performed the whole exome sequencing to identify candidate genes in few *BRCA* negative families. Hamdi et al., performed whole exome sequencing on seven Tunisian families with HBOC and identified four novel BC candidate genes (*MMS19*, *DNAH3*, *POLK* and *KATB6*) [46]. Furthermore, other studies identified by exome sequencing, RCC1 and RAD50 as BC candidate susceptibility genes in Tunisian * BRCA* negative families [47, 48].

## Conclusions

This study assessed the prevalence of germline mutations and identified novel and recurrent mutations for *BRCA1/BRCA2* genes in patients with high risk of BOC from the south region of Tunisia. Giving the high prevalence of pathogenic mutations in *BRCA1/BRCA2 *genes (14.17%), our data will contribute to the establishment of a service dedicated to the genetic screening and to the counseling of the families with high risk of HBOC in Tunisia.

## Supplementary information

**Additional file 1: Figure 1.** Chromatograms showing the wild-type and the mutant DNA sequence together with the IVG or Genome Browser for the following BRCA mutations: a) c.4067_4071 delAAGAA in *BRCA1* gene; b) c. 2338C > T in *BRCA1* gene; c) c.17_20delAAGA in *BRCA2* gene; d)c.1310_1313 delAAGA in *BRCA2* gene and e) c.1796_1800 delCTTAT in *BRCA2* gene.

**Additional file 2: Table 1.** List of Benign/Likely Benign variants identified in the 134 selected patients with breast/ovarian cancer.

## Data Availability

All data generated are included in this article. Raw data are not publically available due to protect the confidentiality of patients, but are available from the corresponding author on request.

## Abbreviations

BRCA1: BReast CAncer susceptibility gene 1
BRCA2: BReast CAncer susceptibility gene 2
BIC: Breast cancer Information Core
BC: Breast cancer
OC: Ovarian cancer
ER: Estrogen receptor
PR: Progesteron receptor
HER2: Human epidermal growth factor receptor 2
TNBC: Triple negative breast cancer
NGS: Next generation sequencing
VUS: Variant of uncertain significance
