# Supplementary figures and images for: Novel and recurrent BRCA1/BRCA2 germline mutations in patients with breast/ovarian cancer: a series from the south of Tunisia
Source: J Transl Med. 2021 Mar 16;19:108. doi: 10.1186/s12967-021-02772-y (PMC7962399; doi:10.1186/s12967-021-02772-y)

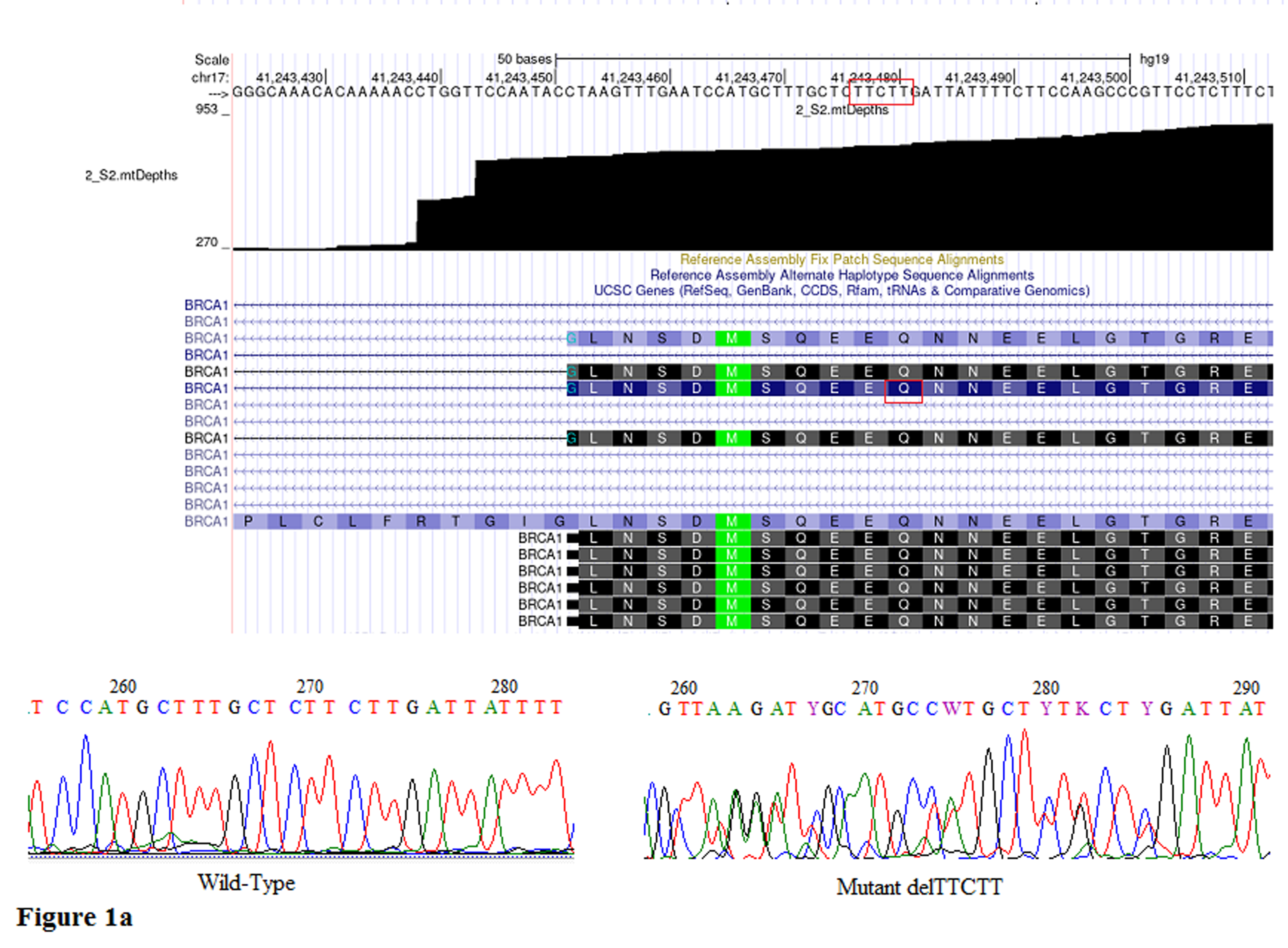

Supplement: Supplementary file 1 — Additional file 1: Figure 1. Chromatograms showing the wild-type and the mutant DNA sequence together with the IVG or Genome Browser for the following BRCA mutations: a) c.4067_4071 delAAGAA in BRCA1 gene; b) c. 2338C > T in BRCA1 gene; c) c.17_20delAAGA in BRCA2 gene; d)c.1310_1313 delAAGA in BRCA2 gene and e) c.1796_1800 delCTTAT in BRCA2 gene. [file 12967_2021_2772_MOESM1_ESM.zip › Figure 1a.png]

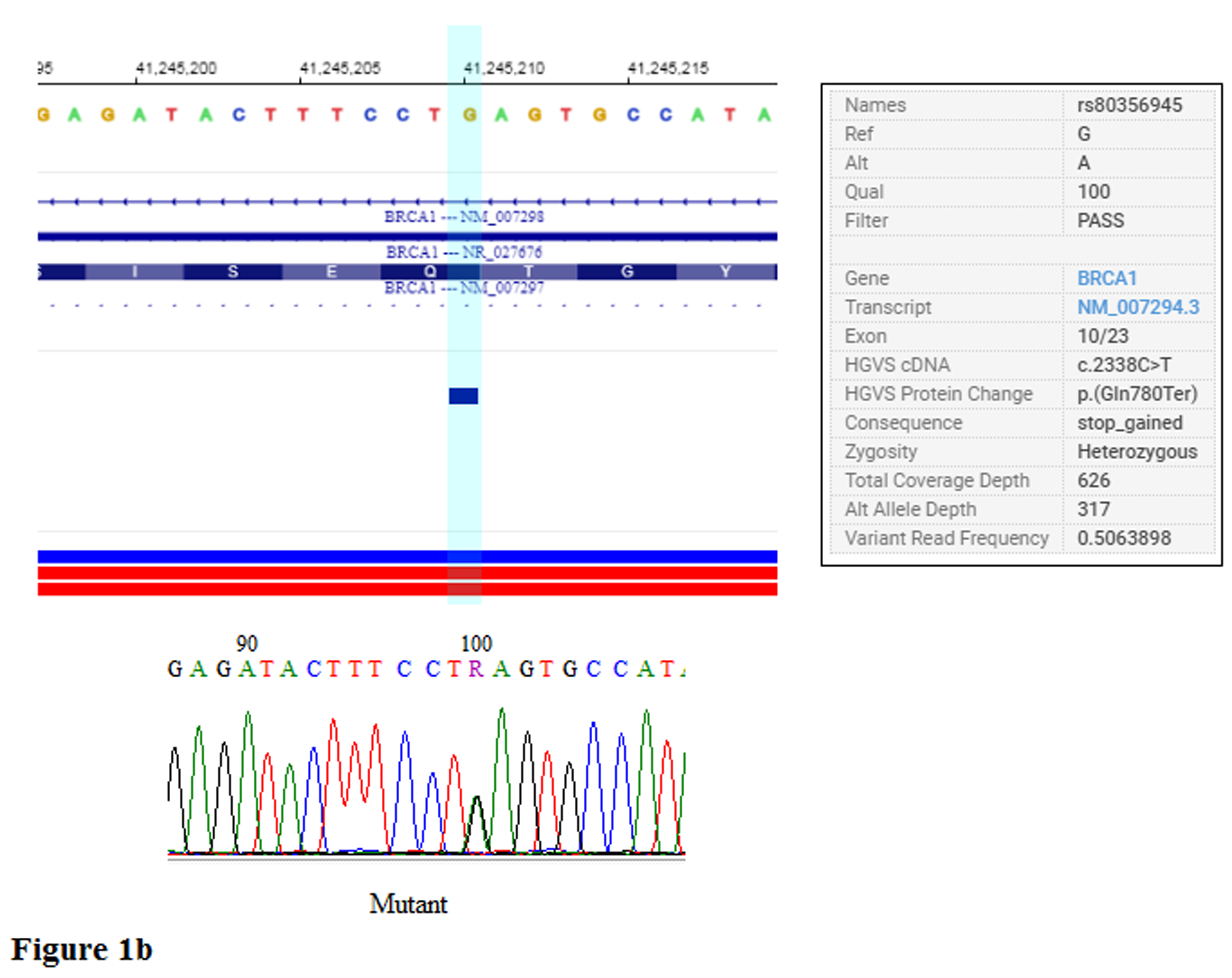

Supplement: Supplementary file 1 — Additional file 1: Figure 1. Chromatograms showing the wild-type and the mutant DNA sequence together with the IVG or Genome Browser for the following BRCA mutations: a) c.4067_4071 delAAGAA in BRCA1 gene; b) c. 2338C > T in BRCA1 gene; c) c.17_20delAAGA in BRCA2 gene; d)c.1310_1313 delAAGA in BRCA2 gene and e) c.1796_1800 delCTTAT in BRCA2 gene. [file 12967_2021_2772_MOESM1_ESM.zip › Figure 1b.png]

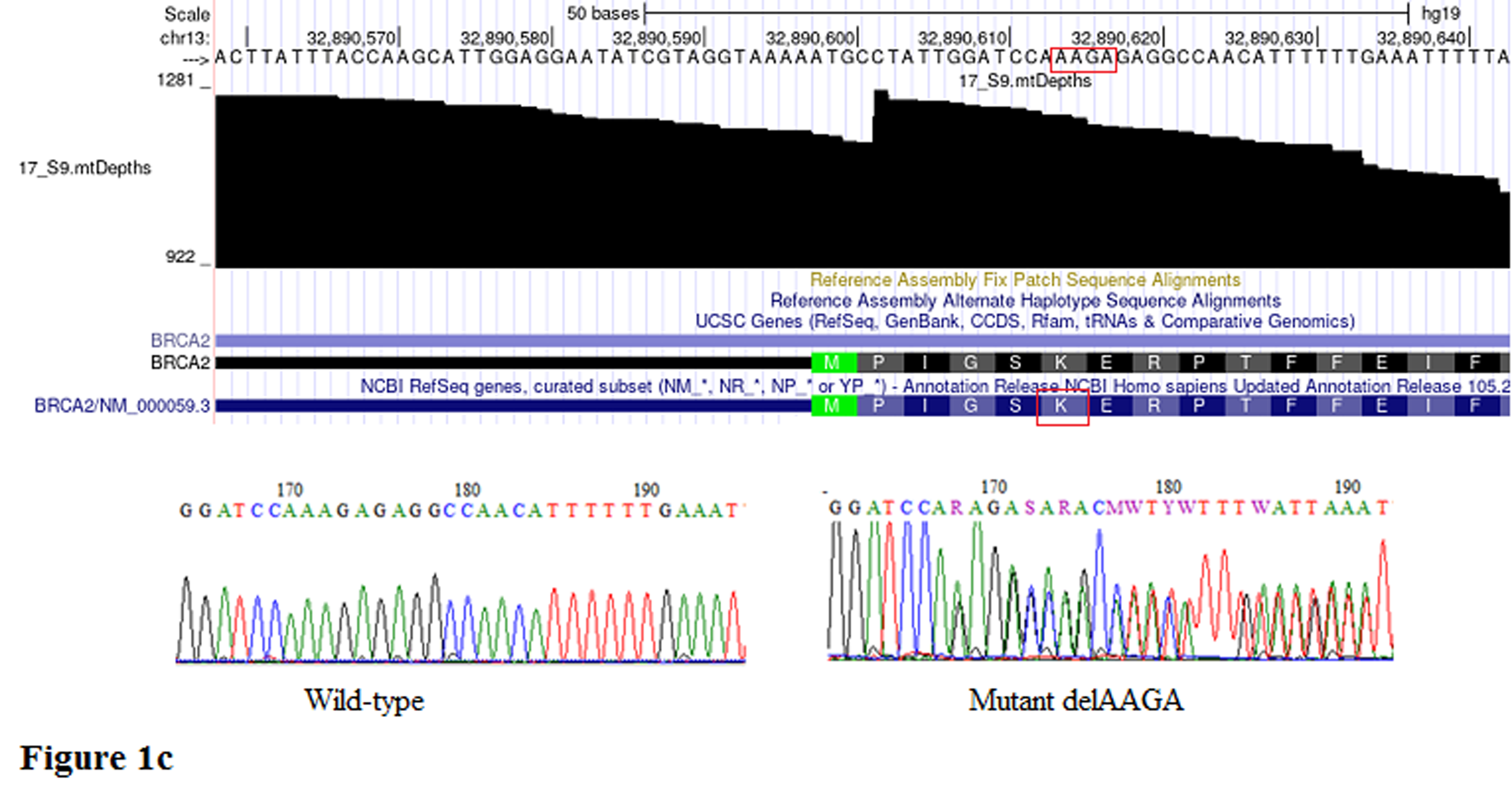

Supplement: Supplementary file 1 — Additional file 1: Figure 1. Chromatograms showing the wild-type and the mutant DNA sequence together with the IVG or Genome Browser for the following BRCA mutations: a) c.4067_4071 delAAGAA in BRCA1 gene; b) c. 2338C > T in BRCA1 gene; c) c.17_20delAAGA in BRCA2 gene; d)c.1310_1313 delAAGA in BRCA2 gene and e) c.1796_1800 delCTTAT in BRCA2 gene. [file 12967_2021_2772_MOESM1_ESM.zip › Figure 1c.png]

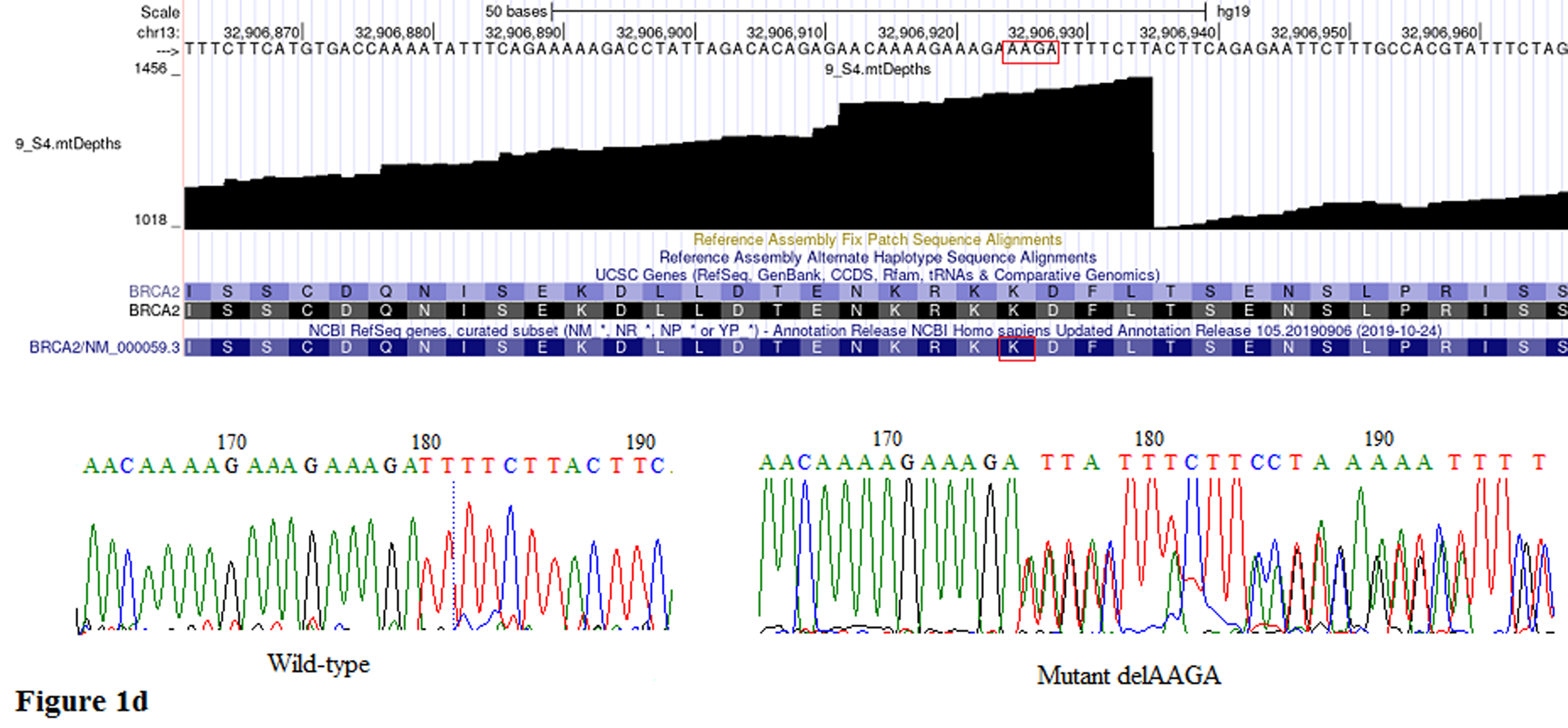

Supplement: Supplementary file 1 — Additional file 1: Figure 1. Chromatograms showing the wild-type and the mutant DNA sequence together with the IVG or Genome Browser for the following BRCA mutations: a) c.4067_4071 delAAGAA in BRCA1 gene; b) c. 2338C > T in BRCA1 gene; c) c.17_20delAAGA in BRCA2 gene; d)c.1310_1313 delAAGA in BRCA2 gene and e) c.1796_1800 delCTTAT in BRCA2 gene. [file 12967_2021_2772_MOESM1_ESM.zip › Figure 1d.png]
