# Supplementary material for: Novel and recurrent BRCA1/BRCA2 germline mutations in patients with breast/ovarian cancer: a series from the south of Tunisia
Source: J Transl Med. 2021 Mar 16;19:108. doi: 10.1186/s12967-021-02772-y (PMC7962399; doi:10.1186/s12967-021-02772-y)
Supplement: Supplementary file 2 — Additional file 2: Table 1. List of Benign/Likely Benign variants identified in the 134 selected patients with breast/ovarian cancer. [file 12967_2021_2772_MOESM2_ESM.docx]

**Table S1:** List of Benign /Likely Benign variants identified in the 134 selected patients with breast/ovarian cancer.

| **GENE** | **Protein variation** | **Coding change** | **Frequency** |
| --- | --- | --- | --- |
| ***BRCA1*** | p.Lys1183Arg | c.3548A>G | 42.53 % (57x) |
|  | p.Glu1038Gly | c.3113A>G | 44.02% (59x) |
|  | p.Pro871Leu | c.2612C>T | 50% (67x) |
|  | p.Leu771Leu | c.2311T>C | 42.53%(57x) |
|  | p.Ser694Ser | c.2082C>T | 39.55%(53x) |
|  | p.Asp693Asn | c.2077G>A | 15.67%(21x) |
|  | p.Ser1634Gly | c.4900A>G | 10.44%(14x) |
|  | p.Ser1436Ser | c.4308T>C | 44.77%(60x) |
|  | p.Ser1040Asn | c.3119G>A | 4.47%(6x) |
|  | p.Ser1613Gly | c.4837A>G | 32.83%(44x) |
|  | p.Phe486Leu | c.1456T>C | 2.23%(3x) |
|  | p.Tyr105Cys | c.314A>G | 0.74%(1x) |
|  | p.Met1008Ile | c.3024G>A | 3.73%(5x) |
|  | p.Gly911Gly | c.2733A>G | 3.73%(5x) |
|  | p.Thr327Thr | c.981A>G | 3.73%(5x) |
|  | p.Arg841Trp | c.2521C>T | 0.74%(1x) |
|  | p.Cys197Cys | c.591C>T | 0.74%(1x) |
|  | p.Asp1546Asn | c.4636G>A | 0.74%(1x) |
|  | p.His1195Arg | c.3584A>G | 0.74%(1x) |
| **TOTAL** | **19** | | |
| ***BRCA2*** | p.Val1269Val | c.3807T>C | 29.10%(39x) |
|  | p.Leu1521Leu | c.4563A>G | 16.41%(22x) |
|  | p.His1561Asn | c.4681C>A | 0.74%(1x) |
|  | p.Val2138Phe | c.6412G>T | 0.74%(1x) |
|  | p.Val2171Val | c.6513G>C | 17.16%(23x) |
|  | p.Val2466Ala | c.7397T>C | 14.92%(20x) |
|  | p.Asn372His | c.1114A>C | 36.56%(49x) |
|  | p.Lys1132Lys | c.3396A>G | 41.04%(55x) |
|  | p.Ser2414Ser | c.7242A>G | 37.31%(50x) |
|  | p.Thr491Ser | c.1472C>G | 1.49%(2x) |
|  | p.Asn 289 His | c.865A>C | 5.97%(8x) |
|  | p.Ser455Ser | c.1365A>G | 6.71%(9x) |
|  | p.His743His | c.2229T>C | 5.22%(7x) |
|  | p.Asn991Asp | c.2971A>G | 5.97%(8x) |
|  | p.Ile1364Leu | c.4090A>C | 0.74%(1x) |
|  | p.Asp935Asn | c.2803G>A | 0.74%(1x) |
|  | Ile3412Val | c.10234A>G | 8.2%(11x) |
|  | p.Thr1915Met | c.5744C>T | 1.49%(2x) |
|  | p.Tyr3098His | c.9292T>C | 1.49%(2x) |
|  | p.Ser2835Pro | c.8503T>C | 1.49%(2x) |
|  | p.Ser1528Ser | c.4584C>T | 0.74%(1x) |
|  | p.Cys1290Tyr | c.3869G>A | 0.74%(1x) |
|  | p.Asp1420Tyr | c.4258G>T | 0.74%(1x) |
|  | p.Gly1771Asp | c.5312G>A | 0.74%(1x) |
|  | p.Asp596Asp | c.1788T>C | 1.49%(2x) |
|  | p.Leu2512Phe | c.7534C>T | 0.74%(1x) |
|  | p.Pro41Leu | c.122C>T | 0.74%(1x) |
|  | p.Ile2944Phe | c.8830A>T | 1.49%(2x) |
|  | p.Asp1902Asn | c.5704G>A | 0.74%(1x) |
|  | p.Ile2521Ile | c.7563C>A | 0.74%(1x) |
|  | p.Leu1356Leu | c.4068G>A | 0.74%(1x) |
| **TOTAL** | **31** | | |
